# Health Tract, No. 167

**Published:** 1863-09

**Authors:** 


HEALTH TRACT, No. 167.

### WORTH REMEMBERING.

1. It is unwise to change to cooler clothing, except when you first get up in the morning.

2. Never ride with your arm or elbow outside any vehicle.

3. The man who attempts to alight from a steam-car while in motion is a fool.

4. In stepping from any wheeled vehicle while in motion, let it be from the rear, and not in front of the wheels; for then, if you fall, the wheels can not run over you.

5. Never attempt to cross a road or street in a hurry in front of a passing vehicle; for if you should stumble or slip, you will be run over. Make up the half-minute lost by waiting until the vehicle has passed, by increased diligence in some other direction.

6. If you want to sleep well at night, avoid sleeping a moment during daylight.

7. It is a miserable economy to save time by robbing yourself of necessary sleep.

8. If you find yourself inclined to wake up at a regular hour in the night and remain awake, you can break up the habit in three days, by getting up as soon as you wake, and not going to sleep again until your usual hour for retiring; or retire two hours later and rise two hours earlier for three days in succession; not sleeping a moment in the day-time.

9. If infants and young children are inclined to be wakeful during the night, or very early in the morning, put them to bed later; and besides, arrange that their day-nap shall be in the forenoon.

10. "Order is heaven's first law," regularity is nature's great rule; hence regularity in eating, sleeping, and exercise, has a very large share in securing a long and healthful life.

11. If you are caught in a drenching rain, or fall in the water, by all means keep in motion sufficiently vigorous to prevent the slightest chilly sensation until you reach the house; then change your clothing with great rapidity before a blazing fire and drink instantly a pint of some hot liquid.

12. To allow the clothing to dry upon you, unless by keeping up vigorous exercise until thoroughly dried, is suicidal.

13. Drop yourself to the ground from the rear of any vehicle when the horses are running away, if you must get out at all.

14. If you are conscious of being in a passion, keep your mouth shut, for words increase it. Many a person has dropped dead in a rage.

15. It does not require a word to make a villainous lie; whatever is intended to deceive or mislead, that is the falsehood. So it does not require a dagger or a bullet to kill a man; the mean slander, a contemptuous shrug, may blast the reputation, and wilt the heart and life away.

16. If a person "faints," place him on his back and let him alone; he wants arterial blood to the head; and it is easier for the heart to throw it there in a horizontal line, than perpendicularly.

17. If you want to get instantly rid of a beastly surfeit, put your finger down your throat until free vomiting, and eat nothing for ten hours.

18. Feel a noble pride in living within your means, then you will not be hustled off to a cheerless hospital in your last sickness.

19. If you would live to purpose, and live long, live industriously, temperately, regularly, all the while maintaining "a conscience void of offense toward God and toward man."

